# The effect of MP29-02* is mediated via bitter taste receptors (TAS2R)

**DOI:** 10.1186/2045-7022-5-S4-P17

**Published:** 2015-06-26

**Authors:** Sandra Ekstedt, Susanna Kumlien Georén, Lars-Olaf Cardell

**Affiliations:** 1 Karolinska Institutet, Division of ENT Diseases, Department of Clinical, Stockholm, Sweden; 2 Karolinska Institutet and Karolinska University Hospital, Division of ENT Diseases, Stockholm, Sweden

## Background

Dymista (MP29-02*) is a novel intranasal formulation of azelastine hydrochloride (AZE) and fluticasone propionate (FP) in an advanced delivery system with a well-documented effect on allergic rhinitis. Its somewhat bitter taste is probably derived from AZE, a potent histamine-H1-receptor antagonist. The taste is sometimes looked upon as a disadvantage. However, it might be that AZE induces some of its beneficial effect through activation of bitter taste receptors. Recent evidence indicates that bitter taste receptors are present in human upper airway mucosa in regions associated with high airflow and particulate deposition. Consequently the MP29-02* effects might be due not only to its anti-histaminic, mast-cell stabilizing, anti-leukotriene, and anti-inflammatory properties but also as due to activation of bitter taste receptors.

## Aim

To explore if MP29-02* has the ability to activate bitter taste receptors using an in-vitro model of isolated murine airways and to probe the potential bitter taste effects of MP29-02* on human nasal epithelial cells.

## Methods

Bitter taste receptor activation was investigated in isolated murine airways incubated in tissue baths. Balb/c mice (male) were used since they are known to have a weak histamine receptor system. MP29-02*, AZE and FP were added cumulatively to tracheal segments pre-contracted with Carbachol. Primary human nasal epithelial cell were cultured in the presence of different concentrations of MP29-02*, AZE and Chloroquine (a bitter taste TAS2R agonist) and evaluated in relation to traditional activation markers, such as VCAM-1 and CD86.

## Results and comments

MP29-02* is a potent dilator of pre-contracted airways, an effect probably mediated by its AZE component. The obtained results make it clear that this is not the result of histamine receptor activation. The relaxant response of MP29-02* and AZE mimics the response induced by the bitter taste TAS2R agonist, Chloroquine. The mechanisms behind bitter taste receptor mediated relaxations are not yet known. The presented experiments rule out the involvement of prostaglandins, cAMP and cGMP (all known to be common pathways for airway dilation) for both MP29-02* and Chloroquine. Furthermore, preliminary data indicate that Dymista® has the ability to trigger nasal epithelia cells in a way that resembles of the effects induced by Azelastin and Chloroquine. There are no known antagonists for bitter taste receptor, but taken together, the presented data supports our notion that MP29-02* is a potent bitter taste agonist, which may contribute to its superior efficacy over AZE and FP observed in clinical trials.

* Dymista

